# The impact of vaccine success and awareness on epidemic dynamics

**DOI:** 10.1063/1.4966945

**Published:** 2016-11-04

**Authors:** Jonq Juang, Yu-Hao Liang

**Affiliations:** 1 Department of Applied Mathematics, and Center of Mathematics Modeling and Scientific Computing, National Chiao Tung University, Hsinchu, Taiwan; 2 Department of Applied Mathematics, National Chiao Tung University, Hsinchu, Taiwan

## Abstract

The role of vaccine success is introduced into an epidemic spreading model consisting of three states: susceptible, infectious, and vaccinated. Moreover, the effect of three types, namely, contact, local, and global, of infection awareness and immunization awareness is also taken into consideration. The model generalizes those considered in Pastor-Satorras and Vespignani [Phys. Rev. E **63**, 066117 (2001)], Pastor-Satorras and Vespignani [Phys. Rev. E **65**, 036104 (2002)], Moreno *et al*. [Eur. Phys. J. B **26**, 521–529 (2002)], Wu *et al*. [Chaos **22**, 013101 (2012)], and Wu *et al*. [Chaos **24**, 023108 (2014)]. Our main results contain the following. First, the epidemic threshold is explicitly obtained. In particular, we show that, for any initial conditions, the epidemic eventually dies out regardless of what other factors are whenever some type of immunization awareness is considered, and vaccination has a perfect success. Moreover, the threshold is independent of the global type of awareness. Second, we compare the effect of contact and local types of awareness on the epidemic thresholds between heterogeneous networks and homogeneous networks. Specifically, we find that the epidemic threshold for the homogeneous network can be lower than that of the heterogeneous network in an intermediate regime for intensity of contact infection awareness while it is higher otherwise. In summary, our results highlight the important and crucial roles of both vaccine success and contact infection awareness on epidemic dynamics.

In this work, we study the effect of vaccine success and the impact of awareness on a general epidemic spreading model consisting of three states: susceptible, infectious, and vaccinated. We obtain two unexpected results. First, if people are aware of the importance of immunization and vaccination is always successful, then, for any initial conditions, the epidemic can be under control even if the spreading rate of the disease is extremely high. Second, it is shown that, although the epidemic threshold is smaller for the heterogeneous network in low and high intensity of contact infection awareness, there is an intermediate regime where the epidemic threshold is in contrast higher for the homogeneous network.

## I. INTRODUCTION

The vaccination is proved to be an effective method to control the spread of epidemic diseases. However, not many theoretical results are done to discuss the impact of vaccine failure, which may go as high as 50%^6^ on epidemic spreading. It is reported^6^ that, although vaccine success has usually been about 85%, success as low as 44% has also been observed. To the best of our knowledge, the first theoretical work to discuss the role of vaccine failure was given in Ref. 7.

In the study of epidemiology, finding ways to prevent the outbreak of an epidemic disease is always an important issue. In the past few decades, several effective immunization strategies have been proposed to minimize the risk of the outbreak of epidemic diseases on complex networks.^2,5,8–16^ Recently, the study of aspects of human responses towards the spread of epidemic diseases has drawn much attention^4,7,17–28^ due to the fact that the change of individual behaviors has an effect on the epidemic dynamics. In the literature,^4,18,29,30^ according to the source of information, awareness is classified into three types, which are termed contact awareness, local awareness, and global awareness. For the first type, it is assumed^4,29^ that individuals with larger contact number in a network are more willing to change their behavior in order to reduce the risk of being infected. The second one is based on personal local information or local infection density.^4,18^ The third one is based on the global infection density in a whole community.^4,31^ The information relating to that may come from national media, e.g., public health authorities. In this work, we shall further differentiate each of the three types of awareness into two cases: infection awareness and immunization awareness. People with infection awareness are more likely to take extra steps to avoid infection, while people with immunization awareness increase likelihood for getting vaccinated. It should also be noted that in heterogeneous networks such as scale-free (SF) ones, the effect of these three types of awareness cannot be separated completely. Such combined effect is also addressed in Ref. 4.

In this work, we consider an epidemic spreading model consisting of three states: susceptible (*S*), infectious (*I*), and vaccinated (*V*), for which the changes of states between *S* and *I* or *S* and *V* take into account the impact of individual awareness and the role of vaccine success. Our main results contain the following. First, the epidemic threshold is explicitly obtained. In particular, if immunization awareness is considered and vaccination has a perfect success, then the epidemic dies out eventually regardless of what other factors are or what initial conditions are. Furthermore, such threshold depends on the contact type of awareness and local infection awareness, and is independent of all the other types of awareness. Such result indeed suggests that the effect of these three types of awareness against the outbreak of an epidemic disease in decreasing order in terms of their importance is contact awareness, local awareness, and global awareness. Second, we compare the epidemic threshold for the homogeneous network, where individuals have roughly the same degree (contact number), and the heterogeneous network, where individuals' degree distribution has a heavy tail. We show that the epidemic threshold for the homogeneous network can be lower than that of the heterogeneous network in an interval *J* of intensity of contact infection awareness, while it is higher otherwise. In certain extreme cases, the interval *J* is empty or infinite.

The organization of the paper is as follows. We introduce our discrete-time epidemic spreading model and its continuous-time version in Section II. The study of the epidemic threshold and the stability of the disease free equilibrium (DFE) on the continuous-time epidemic spreading model are given in Section III. In Section IV, we discuss the effect of heterogeneity of networks on the epidemic threshold. Numerical simulations to support our theoretical results and their other implications are given in Section V. In Section VI, we discuss some of the works related to ours. The summary of our obtained results and the future work is provided in Section VII. In Appendix A, we give the detailed derivation of the continuous-time epidemic spreading model from the discrete-time one. All the proofs of our results are recorded in Appendix B.

## II. EPIDEMIC SPREADING MODEL

In this section, we shall depict the epidemic spreading model under consideration. We begin with the formulation based on probabilistic discrete-time Markov chains. It is assumed that each individual, at each time *t*, is in one of the following three discrete states: infectious (*I*), vaccinated (*V*), and susceptible (*S*), and each could transfer its state at time *t* to a new one at time *t* + 1 through one of the following four different ways: (i) S→I, (ii) S→V, (iii) V→S, and (iv) I→S. Letting $p^{i}(t)$ (respectively, $q^{i}(t)$), i=1,…,N, be the probability that a node *i* is infectious (respectively, vaccinated) at time *t*, our model under consideration reads as follows:

(1) $$\begin{array}{c} p^{i}(t+1)=(1-\gamma )p^{i}(t)+[1-p^{i}(t)-q^{i}(t)]{\text{Prob}}_{i}(S\to I),\\ q^{i}(t+1)=(1-\delta )q^{i}(t)+[1-p^{i}(t)-q^{i}(t)]{\text{Prob}}_{i}(S\to V), \end{array}$$ (1a)

where

$$\begin{array}{c} {\text{Prob}}_{i}(S\to I)\\ =1-{\left[1-\lambda \psi (k_{i})(1-\beta p(t))\left(1-\alpha \frac{s_{i}}{k_{i}}\right)\right]}^{s_{i}}, \end{array}$$ (1b)

$$\begin{array}{c} {\text{Prob}}_{i}(S\to V)\\ =1-\tilde{\psi }(k_{i})\left(1-\tilde{\beta }p(t)\right)\left(1-\tilde{\alpha }\frac{s_{i}}{k_{i}}\right). \end{array}$$ (1c)

Here *k_i_* denotes the degree of a node *i* (i.e., the number of neighbors of a node *i*); *s_i_*, ranging from 0 to *k_i_*, denotes the number of infectious neighbors of a node *i*; and *p*(*t*) denotes the fraction of infectious nodes in the network. For other parameters and terms in (1a)–(1c), their epidemic meanings are explained in the following list.

(I) The parameter γ∈[0,1] in (1a) denotes the *recovering probability* for infectious individuals for the whole time period.
(II) The parameter δ∈[0,1] in (1a) denotes the probability of *vaccine failure* for vaccinated individuals.
(III) The term ${\text{Prob}}_{i}(S\to I)$, defined in (1b), gives the probability that a node *i* changes its state from *S* to *I*. The quantity λ∈[0,1] denotes the *spreading rate* for which an infectious node would actually transmit a disease through an edge connecting to a susceptible node.^32,33^
  (i) The term $\psi (k_{i})$ in (1b) describes the contact awareness to avoid infection for a node *i*,^4,29^ which is to be termed as the *contact infection awareness* in short. Similar usage of the term is to be followed. We assume that a node with a higher degree is aware of the higher risk to be infected and, consequently, it increases its protection and hence reduces the probability of getting infected. Thus, it is assumed that function ψ(k) is decreasing in the degree *k*. In Ref. 4, ψ(k) is chosen to be $k^{-b}$ for some nonnegative constant *b*. The larger the quantity *b* is, the smaller the ψ(k) and the term defined in (1b) is, and hence the less likely a susceptible individual would get infected. The constant *b* is to be termed as the *intensity of contact infection awareness*. Note that, if ψ(k) is independent of *k* (i.e., *b* = 0), then there is no contact infection awareness in the epidemic spreading.
  (ii) The term (1−βp(t)) in (1b) is a decreasing function of *p*(*t*) and *β*. Thus, this term indicates that, as the infectious density *p*(*t*) in the population increases, one increases its protection and hence reduces the probability of getting infected. And so, this term represents the *global infection awareness*.^30,34^ Clearly, such setup indicates that people are made aware of the epidemic disease through national media. Moreover, β∈[0,1] describes the strength of the average risk assessment from global awareness and hence *β* is to be termed as the *intensity of global infection awareness*. For *β* = 0, this means that global infection awareness is not considered in the epidemic spreading.
  (iii) The term $\left(1-\alpha (s_{i}/k_{i})\right)$ in (1b) is a decreasing function of $\left(s_{i}/k_{i}\right)$ and *α*. Thus, this term indicates that as the local infectious density $\left(s_{i}/k_{i}\right)$ of a node *i* increases, one increases its protection and hence reduces the probability of getting infected. And so, this term represents the *local infection awareness*.^5,30,34^ The parameter α∈[0,1] indicates the strength of the average risk assessment from local awareness, and hence *α* is to be termed as the *intensity of local infection awareness*. Note that *α* = 0 means that there is no local infection awareness in the epidemic spreading.
  (iv) The term
    $$\left[1-\lambda \psi (k_{i})\left(1-\beta p(t)\right)\left(1-\alpha \frac{s_{i}}{k_{i}}\right)\right],$$
    represents the probability that a susceptible node *i* will not get infected when it makes a contact with *exactly one* infectious individual. Thus
    $${\left[1-\lambda \psi (k_{i})\left(1-\beta p(t)\right)\left(1-\alpha \frac{s_{i}}{k_{i}}\right)\right]}^{s_{i}},$$
    is the probability that a node *i* will not change its state from susceptible to infectious when it makes contacts with *s_i_* infectious individuals. Thus, ${\text{Prob}}_{i}(S\to I)$, defined in (1b), gives the probability that a node *i* changes its state from susceptible to infectious.
(IV) The term ${\text{Prob}}_{i}(S\to V)$, defined in (1c), gives the probability that a node *i* changes its state from *S* to *V* when the epidemic disease is spreading.^5^
  (i) Similarly, the term $\tilde{\psi }(k_{i})$ in (1c) describes the contact awareness to get immunized, which is to be termed as the *contact immunization awareness* in short. Similar usage of the term is to be followed. We assume that a node with a higher degree is aware of the higher risk to be infected, and it is more likely to get vaccinated to reduce the probability of getting infected. Thus, it is assumed that function $\tilde{\psi }(k)$ is decreasing in *k*. We also set $\tilde{\psi }(k)=k^{-\tilde{b}}$ for some nonnegative constant $\tilde{b}$, where the parameter $\tilde{b}$ is to be termed as the *intensity of contact immunization awareness*. That is, for higher $\tilde{b}$, a susceptible individual tends to have a higher probability of getting vaccinated.
  (ii) The terms $\left(1-\tilde{\beta }p(t)\right)$ and $\tilde{\beta }\in [0,1]$ in (1c) describe the *global immunization awareness* and the *intensity of global immunization awareness*, respectively. Note that ${\text{Prob}}_{i}(S\to V)$ is increasing in $\tilde{\beta }$. Consequently, for higher $\tilde{\beta }$, a susceptible individual is more likely to get vaccinated.
  (iii) The term $\left(1-\tilde{\alpha }(s_{i}/k_{i})\right)$ in (1c) represents the probability that a node *i* changes its state from susceptible to vaccinated due to its local awareness. Note that ${\text{Prob}}_{i}(S\to V)$ is increasing in $\tilde{\alpha }$. Likewise, $\left(1-\tilde{\alpha }(s_{i}/k_{i})\right)$ and the constant $\tilde{\alpha }\in [0,1]$ are called *local immunization awareness* and the *intensity of local immunization awareness*, respectively.

We summarize the epidemic meaning of each parameter and function in Eq. (1) in Table I.

**TABLE I.** The depiction of each parameter and function in epidemic spreading model (1). Note that a node is less (respectively, more) likely to get infected (respectively, immunized) with the increase of intensity *α*, *β*, or *b* (respectively, $\tilde{\alpha },\;\tilde{\beta }$, or $\tilde{b}$).

Description	Parameters	
Spreading rate	*λ*	
Recovering probability	*γ*	
Probability of vaccine failure	*δ*	
Awareness	Infection	Immunization
Intensity of local awareness	*α*	$\tilde{\alpha }$
Intensity of global awareness	*β*	$\tilde{\beta }$
Contact awareness	ψ(k)	$\tilde{\psi }(k)$
Intensity of contact awareness	*b*	$\tilde{b}$

To investigate the effect of the heterogeneity of networks on the epidemic dynamics, we next make a coarse-graining approximation on (1) to derive a continuous-time degree-based mean-field model^1–3,35,36^ by assuming that (i) individuals with the same degree have the same property of dynamical behaviors, (ii) the variable s_i_ could be approximated by its expected value, (iii) the underlying network is uncorrelated, and (iv) the high order terms in (1b)–(1c) are negligible. To begin with, we divide individuals into several distinct groups depending on their degrees *k*. The fraction of the number of individuals with degree *k* is denoted by *P*(*k*) and the corresponding infectious and vaccinated densities among nodes with degree *k* are denoted by $p_{k}(t)$ and $q_{k}(t)$, respectively. The model then reads as follows:

(2) $$\begin{array}{c} {\dot{p}}_{k}(t)=-\gamma p_{k}(t)+[1-p_{k}(t)-q_{k}(t)]\\ \{\lambda \psi (k)\left(1-\beta p(t)\right)\\ \;\Theta (t)\left[k(1-\alpha \Theta (t))-\alpha (1-\Theta (t))\right]\}, \end{array}$$ (2a)

$$\begin{array}{c} {\dot{q}}_{k}(t)=-\delta q_{k}(t)+[1-p_{k}(t)-q_{k}(t)]\\ \left\{1-\tilde{\psi }(k)(1-\tilde{\beta }p(t))\left(1-\tilde{\alpha }\Theta (t)\right)\right\}, \end{array}$$ (2b)

where

$$p(t)=\sum_{k}P(k)p_{k}(t),$$ (2c)

$$\Theta (t)=\frac{\sum_{k}kP(k)p_{k}(t)}{\sum_{k}kP(k)}:=\frac{\sum_{k}kP(k)p_{k}(t)}{\left\langle k\right\rangle }.$$ (2d)

The detailed derivation of the above formula is provided in Appendix A due to its similarity to those in Refs. 4 and 5 and the tediousness.

We end the section by claiming that the epidemic spreading model (2) is well-defined in the sense that if initial conditions $p_{k}(0)$ and $q_{k}(0)$ satisfy $0<p_{k}(0),q_{k}(0)$, and $p_{k}(0)+q_{k}(0)<1$, for all *k*, then $p_{k}(t)$ and $q_{k}(t)$ also satisfy $0<p_{k}(t),q_{k}(t)$, and $p_{k}(t)+q_{k}(t)<1$, for all *k* and *t* > 0. The proof of Proposition 1 is given in Appendix B.

Proposition 1. Define

$$\Delta_{2n}=\{{\left(p_{k},q_{k}\right)}_{1\leq k\leq n}\in {\mathbb{R}}^{2n}:p_{k},\;q_{k}\geq 0\;\text{and}\;p_{k}+q_{k}\leq 1\}.$$

Then $\Delta_{2n}$ is positively an invariant for (2).

## III. STABILITY ANALYSIS AND THE EPIDEMIC THRESHOLD

In this section, we study the epidemic spreading model (2) and compute the threshold for effective spreading rate $\hat{\lambda }\;(:=\lambda /\gamma )$.^1,37^ Our derived results are summarized in the following theorem and the proof is to be provided in Appendix B.

**FIG. 1.**
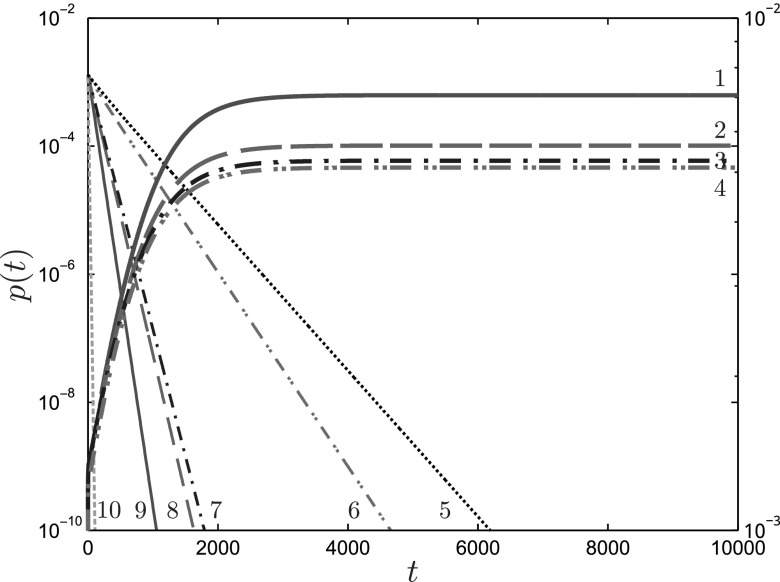
Simulation results in model (2) for the ten cases given in Table II. For case Nos. 1–4, their corresponding time series $(t,p(t)),\;0\leq t\leq 10^{4}$, are plotted with the bold lines and the values of *p*(*t*) are labeled on the right vertical axis of the log scale ranging from $10^{-3}$ to $10^{-2}$. For other cases Nos. 5–10, their corresponding time series $(t,p(t)),\;0\leq t\leq 10^{4}$, are plotted with the fine lines and the values of *p*(*t*) are labeled on the left vertical axis of the log scale, where *p*(*t*) are less than $10^{-10}$ when t≥7000.

**Theorem 1** (see, e.g., Table II and Fig. 1). Consider the epidemic spreading model (2). Then the following two assertions hold. (i) In the case that *δ* = 0 and either ${\tilde{\alpha }}^{2}+{\tilde{\beta }}^{2}>0$ or $\tilde{\psi }(k)\neq 1$, the epidemic dies out. In particular, every solution ${\left(p_{k}(t),\;q_{k}(t)\right)}_{1\leq k\leq n}$ of (2) converges to a disease free equilibrium (DFE) ${\left(0,{\bar{q}}_{k}^{*}\right)}_{1\leq k\leq n}$ for some ${\bar{q}}_{k}^{*}$ in [0,1]. (ii) In other cases, there exists an *epidemic threshold* ${\hat{\lambda }}_{c}$, as given in the following:

$${\hat{\lambda }}_{c}:=\frac{\left\langle k\right\rangle }{\underset{k}{\sum }(1-q_{k}^{*})\psi (k)(k^{2}-\alpha k)P(k)},$$ (3)

where

$$q_{k}^{*}=\{\begin{array}{cc} \frac{\left(1-\tilde{\psi }(k)\right)}{\delta +(1-\tilde{\psi }(k))} & \text{if}\;\delta >0,\\ q_{k}(0) & \text{if}\;\delta =0,\tilde{\alpha }=\tilde{\beta }=0,\tilde{\psi }(k)\equiv 1, \end{array}$$ (4)

such that the epidemic dies out when the *effective spreading rate* $\hat{\lambda }\;(:=\frac{\lambda }{\gamma })$ is smaller than ${\hat{\lambda }}_{c}$; otherwise, the disease breaks out.

**TABLE II.** The table lists the parameters chosen for simulation in Fig. 1. We also record the corresponding epidemic thresholds ${\hat{\lambda }}_{c}$ and final epidemic sizes $p_{\infty }\;(:=\lim_{t\to \infty }p(t))$. The simulation is evaluated for (2) under the degree distribution $P(k)=k^{-r}/c$, k=m,…,M, where γ=2.85, *m* = 2, *M* = 1000, and $c=\sum_{k=m}^{M}k^{-r}$, and with the recovering probability γ=0.15. In the table, the dashed line means that the values of corresponding parameters are chosen to be the same as those given in No. 1.

No.	$\hat{\lambda }$	*α*	*b*	$\tilde{b}$	*δ*	*β*	$\tilde{\beta }$	$\tilde{\alpha }$	${\hat{\lambda }}_{c}$	$p_{\infty }$
1	1.93	0.6	0.8	0.25	0.25	0.1	0.1	0.1	1.90	0.71%
2	…	…	…	…	…	0.6	…	…	…	0.56%
3	…	…	…	…	…	…	0.6	…	…	0.53%
4	…	…	…	…	…	…	…	0.6	…	0.52%
5	1.87	…	…	…	…	…	…	…	…	0
6	…	0.7	…	…	…	…	…	…	1.98	0
7	…	…	0.85	…	…	…	…	…	2.07	0
8	…	…	…	0.3	…	…	…	…	2.05	0
9	…	…	…	…	0.2	…	…	…	2.15	0
10	…	0	0	0	0	0.1	0	0	Nonexistence	0

Remark 1. (i) The implication of the first assertion of the theorem is that if people are aware of the importance of immunization and vaccination has a perfect success, then the epidemic is to die out eventually regardless of what other factors are. (ii) We see clearly, via (3) and (4), that, when δ>0

$${\hat{\lambda }}_{c}=\frac{\left\langle k\right\rangle }{\underset{k}{\sum }\frac{\delta }{\delta +(1-\tilde{\psi }(k))}\psi (k)(k^{2}-\alpha k)P(k)},$$ (5)

which depends on the contact type of awareness (*ψ* and $\tilde{\psi }$), local infection awareness (*α*) and is independent of local immunization awareness ($\tilde{\alpha }$) and global type of awareness (*β* and $\tilde{\beta }$). It should also be noted, though, that $\tilde{\alpha }$, *β*, and $\tilde{\beta }$ play the role on decreasing the final epidemic size ($\underset{t\to \infty }{\lim}p(t)$) when the disease breaks out (see, e.g., Table II and Fig. 1 in Section V).

## IV. EFFECT OF THE DEGREE DISTRIBUTION ON THE EPIDEMIC THRESHOLD

In this section, we consider the impact of awareness on the epidemic threshold ${\hat{\lambda }}_{c}$ as given in (5). Specifically, we assume the contact type of awareness to be given as follows:^4^

$$\psi (k)=k^{-b}\;\text{and}\;\tilde{\psi }(k)=k^{-\tilde{b}}.$$ (6)

It means that individuals with quite a large degree have negligible probability of being infected. Such assumption is also consistent with the strategy of the targeted immunization for the susceptible individuals in the epidemic spreading model,^2^ where individuals with a degree larger than some constant *k*_0_ are much more inclined to take immunization.

### A. Heterogeneous networks

Complex networks describe a wide range of systems in nature and society such as the World Wide Web links, biological networks, and the contact network of individuals.^1^ SF networks,^38^ which are heterogeneous, have frequently been introduced into the epidemic spreading models. Their degree distributions follow a power law $P(k)∼k^{-r}$ and, typically, the exponent r∈(2,3] and k∈[m,M] where *m* and *M* are, respectively, the minimum and maximum of degrees among nodes in SF networks. In this setting, the average of connections ⟨k⟩ is finite but the variance $\langle k^{2}\rangle -{\left\langle k\right\rangle }^{2}$ is infinite as *M* approaches to infinity. Here $\left\langle k^{2}\rangle :=\sum_{k}k^{2}P(k\right)$. Remark that, throughout the paper, we denote by $\left\langle f(k)\rangle =\sum_{k}f(k)P(k\right)$ for any *f*(*k*).

For a SF network, if neither type of awareness is considered, that is, $\alpha =\tilde{\alpha }=\beta =\tilde{\beta }=0$ and $\psi (k)=\tilde{\psi }(k)\equiv 1$, then we have, via (5), that ${\hat{\lambda }}_{c(SF)}=\frac{\left\langle k\right\rangle }{\left\langle k^{2}\right\rangle }$ and hence $\lim_{M\to \infty }{\hat{\lambda }}_{c(SF)}=0$. It implies that the epidemic disease is bound to spread in a SF network with large *M*. However, by increasing the intensity *b* of contact infection awareness, we shall prove in the following theorem that the epidemic can be under control.

**Theorem 2.** Suppose that the probability *δ* of vaccine failure is positive. Then the epidemic threshold ${\hat{\lambda }}_{c(SF)}$ for a SF network with the exponent r∈(2,3] and k∈[m,M] tends to be 0 as *M* approaches to ∞ if and only if b≤3−r.

The above theorem indicates that the intensity *b* of contact infection awareness plays a critical role in determining the outbreak of the epidemic disease in SF networks. When the vaccine success is not perfect and the intensity *b* of contact infection awareness is low, the epidemic threshold ${\hat{\lambda }}_{c}$ becomes zero in the limit. As a result, even for extremely low effective spreading rates, the disease would be able to diffuse through the population and prevail in the SF networks. However, by sufficiently increasing the intensity *b* of contact infection awareness, the epidemic threshold ${\hat{\lambda }}_{c}$ then in the limit becomes a positive finite value.

### B. Homogeneous networks

In this subsection, we consider the epidemic disease spreading in the homogeneous network and compare its epidemic threshold with that of the heterogeneous network (specifically, the SF network) under the assumption that two networks have the same average degree number ⟨k⟩. Contrary to the heterogeneous network that owns a long-tail degree distribution, another wide class of networks has exponentially bounded degree fluctuations and each node in the network has roughly the same number of links, k≃⟨k⟩. Networks of this property are called the homogeneous networks. Paradigmatic homogenous networks are the Erdös-Rényi random graphs and the Watts-Strogatz (WS) small-world models. For simplicity, we assume herein that all nodes in the homogeneous network have exactly the same degree. Then, via (5), we have that the corresponding epidemic threshold is

$${\hat{\lambda }}_{c}=\frac{\left\langle k\right\rangle }{\frac{\delta }{\delta +[1-{\left\langle k\right\rangle }^{-\tilde{b}}]}{\left\langle k\right\rangle }^{-b}\left[{\left\langle k\right\rangle }^{2}-\alpha \langle k\rangle \right]}=:{\hat{\lambda }}_{c(Homo)}.$$ (7)

Note that, when neither type of awareness is considered, that is, $\tilde{b}=b=\alpha =0$, we have, via (7), that ${\hat{\lambda }}_{c(Homo)}=\langle k\rangle /{\left\langle k\right\rangle }^{2}$. Consequently

$${\hat{\lambda }}_{c(SF)}\left(=\langle k\rangle /\langle k^{2}\rangle \right)<{\hat{\lambda }}_{c(Homo)}\left(=\langle k\rangle /{\left\langle k\right\rangle }^{2}\right),$$ (8)

which implies that the epidemic disease is easier to break out in the heterogeneous network than in the homogeneous network. However, when awareness is taken into account, some more complicated and interesting results concerning the epidemic threshold ${\hat{\lambda }}_{c}$ can be observed. We will state our finding in the following theorems. The detailed proofs of these results, making use of Jesen's inequality and some properties of log-convex functions, are recorded in Appendix B.

**Theorem 3**. Suppose $\tilde{b}=0,\;\delta >0$ and k∈[m,M]. Then, for any α∈[0,1), there exists some $b_{2}\;(=b_{2}(\alpha ,m,M))\in [2,\frac{2}{1-\alpha })$ such that

$$\{\begin{array}{cc} {\hat{\lambda }}_{c(SF)}<{\hat{\lambda }}_{c(Homo)} & \text{if}\;b\in [0,1)\cup (b_{2},\infty ),\\ {\hat{\lambda }}_{c(SF)}={\hat{\lambda }}_{c(Homo)} & \text{if}\;b=1\text{or}b_{2},\\ {\hat{\lambda }}_{c(SF)}>{\hat{\lambda }}_{c(Homo)} & \text{if}\;b\in (1,b_{2})=:J_{0,\alpha ,\delta ,m}. \end{array}$$

Moreover, $b_{2}(\alpha ,m,M)$ is strictly increasing in *α* and satisfies $\frac{\langle k^{2-b_{2}}\rangle -{\left\langle k\right\rangle }^{2-b_{2}}}{\langle k^{1-b_{2}}\rangle -{\left\langle k\right\rangle }^{1-b_{2}}}=\alpha$.

The theorem is essentially amount to saying that, for $\tilde{b}=0$, there exists a nonempty and finite interval $J=(1,b_{2})$ for which the epidemic disease is easier to break out in the homogeneous network than in the SF network provided that the intensity *b* of contact infection awareness lies in *J*. On the other hand, if $b\notin [1,b_{2}]$, then the epidemic disease is easier to break out in the SF network.

In the next theorem, we show that, if the intensity $\tilde{b}$ of contact immunization awareness is nonzero, then the corresponding interval *J* could be empty, nonempty and finite, or infinite.

**Theorem 4**. Suppose $\tilde{b}>0,\;\delta >0$ and k∈[m,M]. Then, for any α∈[0,1], there exists at most one open and connected interval $J_{\tilde{b},\alpha ,\delta ,m}$ such that

$${\hat{\lambda }}_{c(SF)}>{\hat{\lambda }}_{c(Homo)}\;\text{if}\;\text{and}\;\text{only}\;\text{if}\;b\in J_{\tilde{b},\alpha ,\delta ,m}.$$

Moreover, ${\hat{\lambda }}_{c(SF)}={\hat{\lambda }}_{c(Homo)}$ if and only if *b* satisfies

$$\frac{\left\langle \frac{\delta k^{2-b}}{\delta +1-k^{-\tilde{b}}}\right\rangle -\frac{\delta }{\delta +1-{\left\langle k\right\rangle }^{-\tilde{b}}}{\left\langle k\right\rangle }^{2-b}}{\left\langle \frac{\delta k^{1-b}}{\delta +1-k^{-\tilde{b}}}\right\rangle -\frac{\delta }{\delta +1-{\left\langle k\right\rangle }^{-\tilde{b}}}{\left\langle k\right\rangle }^{1-b}}=\alpha .$$

Note that $J_{\tilde{b},\alpha ,\delta ,m}$ could be either of the form (*a*, *b*), 0<a<b<∞, or of the form (a,∞), or the empty set.

For *m*, the minimum degree among nodes in the SF network, sufficiently large and δ≠0, we are able to show that $J_{\tilde{b},\alpha ,\delta ,m}$ must be an open, connected, nonempty, and finite interval. In particular, we prove that, given any set of parameters $\left(b,\tilde{b},\alpha )\in {\mathbb{R}}^{+}\times {\mathbb{R}}^{+}\times [0,1\right]$, its corresponding sign of ${\hat{\lambda }}_{c(SF)}-{\hat{\lambda }}_{c(Homo)}$ can be determined as long as *m* is sufficiently large and δ≠0. The above mentioned result is recorded in the following theorem.

**Theorem 5**. Consider $\tilde{b},\;\delta >0$ and let *A* be the union of the sets *A_i_*, i=1,2,…,5, where $A_{1}=\left(\left[0,1)\cup (2,\infty \right)\right)\times {\mathbb{R}}^{+}\times [0,1]$, $A_{2}=\{1\}\times (1,\infty )\times [0,1]$, $A_{3}=\{1,2\}\times \{1\}\times [0,\frac{1}{\delta +1})$, $A_{4}=\{2\}\times (1,\infty )\times \{0\}$, $A_{5}=\{2\}\times (0,1)\times [0,1]$, and $B=\{1,2\}\times \{1\}\times \{\frac{1}{\delta +1}\}$. Here ${\mathbb{R}}^{+}=[0,\infty )$. Then the following holds.

(i) For any set of parameters $(b,\tilde{b},\alpha )\in A$, there exists some positive integer *m*_0_ such that ${\hat{\lambda }}_{c(SF)}<{\hat{\lambda }}_{c(Homo)}$ whenever the minimum degree *m* in the SF network is greater than or equal to *m*_0_. In particular, when $(b,\tilde{b},\alpha )\in A_{3}\cup A_{4}$, we have that $m_{0}=1$.
(ii) For any set of parameters $(b,\tilde{b},\alpha )\in B$, ${\hat{\lambda }}_{c(SF)}={\hat{\lambda }}_{c(Homo)}$.
(iii) For any set of parameters $(b,\tilde{b},\alpha )/A\cup B$, there exists some positive integer *m*_0_ such that ${\hat{\lambda }}_{c(SF)}>{\hat{\lambda }}_{c(Homo)}$ whenever the minimum degree *m* in the SF network is greater than or equal to *m*_0_. In particular, when *b* = 1 or 2, $\tilde{b}=1$ and $\alpha >\frac{1}{\delta +1}$, we have that $m_{0}=1$.

Above theorem implies that, for any given $\tilde{b}\geq 0,\;0\leq \alpha \leq 1$ and δ>0, the interval $J_{\tilde{b},\alpha ,\delta ,m}$ is open, connected, *nonempty*, and finite whenever *m* is sufficiently large. Indeed, for any b∈(0,1)∪(2,∞) (respectively, b∈(1,2)), there exists some integer *m*_0_ such that $b\notin J_{\tilde{b},\alpha ,\delta ,m}$ (respectively, $b\in J_{\tilde{b},\alpha ,\delta ,m}$) whenever $m\geq m_{0}$ since $(b,\tilde{b},\alpha )\in A_{1}$ (respectively, $(b,\tilde{b},\alpha )\notin A\cup B$).

## V. SIMULATIONS

In the first part of this section, we illustrate some numerical simulations for (coarse-graining) epidemic spreading model (2) to verify that the observations made in Remark 1 (ii) are indeed true. To see this, we set, in (2), $P(k)=k^{-r}/c,k=m,\ldots ,M$, where *r* = 2.85, *m* = 2, *M* = 1000, and $c=\sum_{k=m}^{M}k^{-r}$, while all other parameters are treated as testing variables. The simulation results in model (2) are provided in Table II and Fig. 1. In Table II, we record the parameters used for simulation, epidemic thresholds ${\hat{\lambda }}_{c}$ computed by (5) and the finial epidemic sizes $p_{\infty }\;(:=\lim_{t\to \infty }p(t))$ from the simulation. It can be observed that, for epidemic spreading model (2), if the parameters are chosen as those in No. 1 (respectively, No. 5) in Table II, then $\hat{\lambda }\;(=1.93)>{\hat{\lambda }}_{c}\;(\approx 1.90)$ (respectively, $\hat{\lambda }\;(=1.87)<{\hat{\lambda }}_{c}\;(\approx 1.90)$). Consequently, we have that the epidemic disease breaks out (respectively, dies out) and its corresponding $p_{\infty }\approx 0.71\%$. Meanwhile, if we increase the intensity $\tilde{b}$, *b*, or *α* of awareness or decrease the probability *δ* of vaccine failure, then the outbreak of the epidemic disease can be prevented (see Nos. 6–9 in Table II). However, if we increase the intensity *β*, $\tilde{\beta }$, or $\tilde{\alpha }$ of awareness, then it cannot prevent the epidemic outbreak but helps to reduce the final epidemic size (see Nos. 2–4 in Table II). We also point out that, if the vaccine success is perfect and immunization awareness is introduced, then the epidemic would eventually die out (see No. 10 in Table II). The time series simulation for the cases in Table II are given in Fig. 1.

We next aim to compare the size of ${\hat{\lambda }}_{c(SF)}$ and ${\hat{\lambda }}_{c(Homo)}$ claimed in Theorems 4 and 5. To this end, we fix α=0.6 and $\tilde{b}=\delta =0.25$. In addition, to compute ${\hat{\lambda }}_{c(SF)}$, we set $P(k)=k^{-r}/c,\;k=m,\ldots ,M$, where *r* = 2.85, *M* = 1000, and $c=\sum_{k=m}^{M}k^{-r}$ in (5), and, to compute ${\hat{\lambda }}_{c(Homo)}$, we set $\langle k\rangle =\sum_{k=m}^{M}k^{1-r}/c$ in (7). Here, the parameters *m* and *b* are treated as the testing ones, and their choices and their corresponding epidemic thresholds ${\hat{\lambda }}_{c}$ are given in Table III. In Table III, we see that the sign of ${\hat{\lambda }}_{c(SF)}-{\hat{\lambda }}_{c(Homo)}$ changes twice from case 1 to 3 as *b* varies from *b* = 0.1 to *b* = 0.8 to *b* = 2.8. This indicates the existence of the interval $J_{\tilde{b},\alpha ,\delta ,m}$ as claimed in Theorem 4. On the other hand, the computed epidemic thresholds ${\hat{\lambda }}_{c}$ for cases 2, 4, and 5 seem to suggest that ${\hat{\lambda }}_{c(SF)}<{\hat{\lambda }}_{c(Homo)}$ whenever $m\geq 2\;(=m_{0})$ as claimed in Theorem 5(i).

**TABLE III.** The table gives the values of epidemic thresholds ${\hat{\lambda }}_{c(SF)}$ and ${\hat{\lambda }}_{c(Homo)}$ with fixed parameters α=0.6 and $\tilde{b}=\delta =0.25$ and varying parameters *m* and *b*.

Case	*m*	*b*	${\hat{\lambda }}_{c}$
1	1	0.1	$\;{\hat{\lambda }}_{c(SF)}\;(\approx 0.65)<{\hat{\lambda }}_{c(Homo)}\;(\approx 1.64)$
2	1	0.8	$\;{\hat{\lambda }}_{c(SF)}\;(\approx 2.19)>{\hat{\lambda }}_{c(Homo)}\;(\approx 2.14)$
3	1	2.8	$\;{\hat{\lambda }}_{c(SF)}\;(\approx 4.05)<{\hat{\lambda }}_{c(Homo)}\;(\approx 4.59)$
4	2	0.8	$\;{\hat{\lambda }}_{c(SF)}\;(\approx 1.90)<{\hat{\lambda }}_{c(Homo)}\;(\approx 1.95)$
5	3	0.8	$\;{\hat{\lambda }}_{c(SF)}\;(\approx 1.84)<{\hat{\lambda }}_{c(Homo)}\;(\approx 1.91)$

For clarity, we summarize the results of Theorems 3–5 and our observations from the simulations in the following Table IV. Let $J_{\tilde{b},\alpha ,\delta ,m}$ be the set of the parameters *b* so that ${\hat{\lambda }}_{c(SF)}>{\hat{\lambda }}_{c(Homo)}$. The table gives the range of the parameters $\left(\tilde{b},\alpha ,\delta ,m\right)$ for which their corresponding $J_{\tilde{b},\alpha ,\delta ,m}$ is finite, infinite, or empty. The assertions made in the second row in Table IV is rigorous. The assertions in the third and fourth rows are based on numerical simulations. It is clear then that $J_{\tilde{b},\alpha ,\delta ,m}$ being nonempty and finite is generic. Consequently, the epidemic disease is easier (respectively, harder) to break out in the homogeneous network than in the heterogeneous network whenever the intensity of contact infection awareness is neither too low nor too high (respectively, intermediate).

**TABLE IV.** Three possible cases for interval $J_{\tilde{b},\alpha ,\delta ,m}$ and their corresponding range of parameters. Here $J_{\tilde{b},\alpha ,\delta ,m}$ is the collection of intensity *b* of contact infection awareness so that ${\hat{\lambda }}_{c(SF)}>{\hat{\lambda }}_{c(Homo)}$.

$J_{\tilde{b},\alpha ,\delta ,m}$	Range of parameters
$0<|J_{\tilde{b},\alpha ,\delta ,m}|<\infty$	$\left(\tilde{b}=0,\;0\leq \alpha <1,\;0<\delta \leq 1,\;m\in \mathbb{N}\right)$
	$\left(\tilde{b}>0,\;0\leq \alpha \leq 1,\;0<\delta \leq 1,\;m\geq m_{0}\right)$
$|J_{\tilde{b},\alpha ,\delta ,m}|=\infty$	$\left(\tilde{b}\geq 0,\;\alpha =1,\;0<\delta \leq 1,\;m=1\right)$
$|J_{\tilde{b},\alpha ,\delta ,m}|=Ø$	$\left(\tilde{b}>0,\alpha \ll 1,0<\delta \ll 1,m\leq m_{1}\right)$

## VI. RELATED MODELS

In this section, we demonstrate that our model (2) is a generalized model for those considered in Refs. 1–5. Indeed, (i) if there is no immunization awareness, i.e., $\tilde{\alpha }=\tilde{\beta }=0,\;\tilde{\psi }(k)\equiv 1$ and the vaccine success is perfect, i.e., *δ* = 0, and if, in addition, we let $q_{k}(0)=0$, then the model becomes the one considered in Ref. 4. Moreover, ${\hat{\lambda }}_{c}$ in (3) is reduced to the following:

$${\hat{\lambda }}_{c}=\frac{\left\langle k\right\rangle }{\sum_{k}\psi (k)(k^{2}-\alpha k)P(k)}=\frac{\left\langle k\right\rangle }{\left\langle k^{2}\psi (k)\rangle -\alpha \langle k\psi (k)\right\rangle },$$

which is the one obtained in [Eq. (11), Ref. 4]. (ii) For model without considering infection awareness, i.e., α=β=0, ψ(k)≡1, we have that

$${\dot{p}}_{k}(t)=-\gamma p_{k}(t)+\lambda k\Theta (t)[1-p_{k}(t)],$$

which is the model considered in Refs. 1–3. The corresponding epidemic thresholds ${\hat{\lambda }}_{c(SF)}$ and ${\hat{\lambda }}_{c(Homo)}$ are computed as in (8). They are in agreement with those given in Refs. 1–3 and 39. (iii) If only local immunization awareness is considered, i.e., $\tilde{\alpha }>0,\;\alpha =\beta =\tilde{\beta }=0$ and $\psi (k)=\tilde{\psi }(k)\equiv 1$, then our discrete-time version of the epidemic spreading model given in (A6) with *h* = 1 agrees with that considered in Ref. 5.

## VII. CONCLUSION

We proposed an epidemic spreading model including the element of vaccine failure and three types of infection awareness and immunization awareness. Our results generalize the established results on reduced forms of the model presented here. We also find that the epidemic threshold for the homogeneous network can be lower than that of the heterogeneous network provided that the intensity of contact infection awareness lies in an intermediate regime. It is of interest to study the effect of vaccine failure and awareness on cooperative^40–42^ or competitive^43,44^ disease dynamics.

## APPENDIX A: THE DERIVATION OF THE EPIDEMIC SPREADING MODEL (2)

In this section, we give the detailed derivation of the epidemic spreading model (2) from (1). We first make some coarse-graining approximations on (1) to get the discrete-time heterogeneous mean-field model of it. To this end, we divide the individuals into several distinct groups depending on their degrees *k*. Denote by *P*(*k*), the fraction of the number of individuals with degree *k*, and $p_{k}(t)$ and $q_{k}(t)$ by the corresponding infectious and vaccinated densities among nodes with degree *k*, respectively. Then it is assumed that each individual with the same degree has the same property of dynamical behaviors. To be more precise, we assume that, for each node *i* in the subgroup with degree *k*

$$k_{i}\equiv k,\;s_{i}\equiv s,\;p^{i}\equiv p_{k}\text{and}\;q^{i}\equiv q_{k}.$$

It follows that (1b) and (1c) become, respectively:

$$\begin{array}{c} {\text{Prob}}_{i}(S\to I)=1-{\left[1-\lambda \psi (k)\left(1-\alpha \frac{s}{k}\right)\left(1-\beta p(t)\right)\right]}^{s}\\ =:1-\omega (s,k),\\ {\text{Prob}}_{i}(S\to V)=1-\tilde{\psi }(k)\left(1-\tilde{\alpha }\frac{s}{k}\right)\left(1-\tilde{\beta }p(t)\right)\\ =:\tilde{\omega }(s,k), \end{array}$$

where $p(t)=\sum_{j=1}^{N}p^{j}(t)=\sum_{k}P(k)p_{k}(t)$. Then we use the expected value of variable *s* to get an approximation of it. That is, we assume that

$$\begin{array}{c} {\text{Prob}}_{i}(S\to I)\approx E[1-\omega (s,k)]=\sum_{s=1}^{k}[1-\omega (s,k)]B(s,k),\\ {\text{Prob}}_{i}(S\to V)\approx E[\tilde{\omega }(s,k)]=\sum_{s=1}^{k}\tilde{\omega }(s,k)B(s,k). \end{array}$$ (A1)

Here *B*(*s*, *k*) is the probability that a node with degree *k* has exactly *s* infectious neighbors, and it is assumed to satisfy the binomial distribution.^45^ That is

$$B(s,k)=\left(\begin{array}{c} k\\ s \end{array}\right){\left[\Theta (t)\right]}^{s}{\left[1-\Theta (t)\right]}^{k-s},$$ (A2)

where Θ(t) is the probability of a randomly selected link pointing to an infectious individual, and it is assumed to be^1,37^

$$\Theta (t)=\sum_{k}\frac{kP(k)}{\left\langle k\right\rangle }p_{k}(t)=\frac{\sum_{k}kP(k)p_{k}(t)}{\left\langle k\right\rangle },$$ (A3)

as defined in (2d). Herein, we assume that the underlying network is uncorrelated.

Following by the assumptions of (A1), (A2), and (A3), we compute that:

$$\begin{array}{c} {\text{Prob}}_{i}(S\to I)\\ \approx \sum_{s=0}^{k}\left\{1-{\left[1-\lambda \psi (k)\left(1-\alpha \frac{s}{k}\right)\left(1-\beta p(t)\right)\right]}^{s}\right\}B(s,k)\\ =1-\sum_{s=0}^{k}{\left[1-\lambda \psi (k)\left(1-\alpha \frac{s}{k}\right)\left(1-\beta p(t)\right)\right]}^{s}B(s,k)\\ \approx 1-\sum_{s=0}^{k}\left[1-s\lambda \psi (k)\left(1-\alpha \frac{s}{k}\right)\left(1-\beta p(t)\right)\right]B(s,k)\\ =\lambda \psi (k)\left(1-\beta p(t)\right)\sum_{s=0}^{k}s\left(1-\alpha \frac{s}{k}\right)B(s,k)\\ =\lambda \psi (k)\left(1-\beta p(t)\right)\left(E[s]-\frac{\alpha }{k}E[s^{2}]\right)\\ =\lambda \psi (k)\left(1-\beta p(t)\right)\Theta (t)[k(1-\alpha \Theta (t))-\alpha (1-\Theta (t))], \end{array}$$ (A4)

since E[s]=kΘ and $E[s^{2}]=k^{2}\Theta^{2}+k\Theta -k\Theta^{2}$. Similarly

$$\begin{array}{c} {\text{Prob}}_{i}(S\to V)\;\;\\ \approx \sum_{s=0}^{k}\left[1-\tilde{\psi }(k)\left(1-\tilde{\alpha }\frac{s}{k}\right)\left(1-\tilde{\beta }p(t)\right)\right]B(s,k)\;\;\;\;\\ =\left[1-\tilde{\psi }(k)\left(1-\tilde{\beta }p(t)\right)\left(1-\tilde{\alpha }\Theta (t)\right)\right]. \end{array}$$ (A5)

Next, to derive the continuous version of the epidemic spreading model (2), we first shorten the time interval for the iterations from [t,t+1) to [t,t+h). In the time interval [t,t+h), we assume that the probability of recovering and vaccine failure to be *γh* and *δh*, respectively. Similarly, we assume that ${\text{Prob}}_{i,h}(S\to I)=h{\text{Prob}}_{i}(S\to I)$ and ${\text{Prob}}_{i,h}(S\to V)=h{\text{Prob}}_{i}(S\to V)$, which means that the probability that an individual changes its state depends linearly on the length of exposure. Then, by (1a) and letting $p^{i}\equiv p_{k}$ and $q^{i}\equiv q_{k}$, we have that the discrete-time heterogeneous mean-field model of (1) reads as follows:

$$\begin{array}{c} p_{k}(t+h)=(1-\gamma h)p_{k}(t)\\ \;+h[1-p_{k}(t)-q_{k}(t)]{\text{Prob}}_{i}(S\to I),\\ q_{k}(t+h)=(1-\delta h)q_{k}(t)\\ \;+h[1-p_{k}(t)-q_{k}(t)]{\text{Prob}}_{i}(S\to V). \end{array}$$ (A6)

Using (A6), (A4), and (A5) and letting *h* tends to be 0, we arrive at the continuous version of the epidemic spreading model as given in (2).

## APPENDIX B: PROOFS IN THE MAIN TEXT

In this section, we give detailed proofs of Proposition 1, and Theorems 1–5.

*Proof of Proposition 1*. To show that $\Delta_{2n}$ is positively invariant for (2), it suffices to claim that the vector field defined by (2) is tangent or points into $\Delta_{2n}$ on the boundary $\partial \Delta_{2n}$ of $\Delta_{2n}$. Clearly, $(p_{k},q_{k})\in \partial \Delta_{2n}$ if $(p_{k},q_{k})\in \Delta_{2n}$ and either $p_{k_{0}}=0,\;q_{k_{0}}=0$ or $p_{k_{0}}+q_{k_{0}}=1$ for some *k*_0_. Note, via (2), that we have ${\dot{p}}_{k_{0}}\geq 0$ (respectively, ${\dot{q}}_{k_{0}}\geq 0$) whenever $p_{k_{0}}=0$ (respectively, $q_{k_{0}}=0$). Similarly, ${\dot{p}}_{k_{0}}+{\dot{q}}_{k_{0}}=-\gamma p_{k_{0}}-\delta q_{k_{0}}\leq 0$ provided that $p_{k_{0}}+q_{k_{0}}=1$. The proof of the proposition is just completed. □

*Proof of Theorem 1*. Let $\left(p^{*},q^{*}\right)$ be a DFE of (2), where $p^{*}:=(p_{1}^{*},p_{2}^{*},\ldots ,p_{n}^{*})=(0,0,\ldots ,0)\;(=:0)$ and $q^{*}:=(q_{1}^{*},q_{2}^{*},\ldots ,q_{n}^{*})\in {\left[0,1\right]}^{n}$. Then some direct computation from (2) yields that $q_{k}^{*}$ satisfies $-[\delta +(1-\tilde{\psi }(k))]q_{k}^{*}+(1-\tilde{\psi }(k))=0$. Hence, (i) if *δ* = 0 and $\tilde{\psi }(k)=1$, then $q_{k}^{*}$ can be any arbitrary value in [0,1], while (ii) if δ>0 or $\tilde{\psi }(k)\neq 1$, then $q_{k}^{*}=\frac{\left(1-\tilde{\psi }(k)\right)}{\delta +(1-\tilde{\psi }(k))}$, as defined in the first equation of (4).

To see the first assertion of the theorem, let $p=(p_{1},p_{2},\ldots ,p_{n})$ and $q=(q_{1},q_{2},\ldots ,q_{n})$. Consider the Lyapunov candidate function $V(p,q)=\frac{1}{2}\sum_{k}{\left(q_{k}-1\right)}^{2}$. Then $\dot{V}(p,q)=\sum_{k}\left(q_{k}-1\right){\dot{q}}_{k}\leq 0$ since, as can been seen in Eq. (2), ${\dot{q}}_{k}(t)\geq 0$ when *δ* = 0, and $q_{k}\leq 1$ by Proposition 1. By LaSalle's invariant principle, all solutions of (2) approach the largest invariant *S* of $\left\{(p,q)\in \Delta_{2n}:\dot{V}(p,q)=0\right\}$. Clearly, when *δ* = 0, $\dot{V}=0$ if and only if $p_{k}+q_{k}=1$ or p=0 by (2) and the definitions of *p* and Θ since either ${\tilde{\alpha }}^{2}+{\tilde{\beta }}^{2}>0$ or $\tilde{\psi }(k)\neq 1$. However, when $p_{k}+q_{k}=1,{\dot{p}}_{k}\;+{\dot{q}}_{k}=-\gamma p_{k}\leq 0$ and the equality holds if and only if *p_k_* = 0. It implies that *p_k_* = 0 for points in *S*. Thus, for every solution ${\left(p_{k}(t),\;q_{k}(t)\right)}_{1\leq k\leq n}$ of (2), $p_{k}(t)$ converges to 0 for each *k*. Moreover, since $q_{k}(t)$ is increasing and bounded above, $q_{k}(t)$ also converges to some fixed point ${\bar{q}}_{k}^{*}$ in [0,1].

We next prove the second assertion of the theorem. From (2), it can be easily seen that

$$\begin{array}{c} {\dot{p}}_{k}(t)=-\gamma p_{k}(t)+\lambda \psi (k)(k-\alpha )[1-q_{k}(t)]\Theta (t)+o(p(t)),\\ {\dot{q}}_{k}(t)=-\delta q_{k}(t)+[1-p_{k}(t)-q_{k}(t)]\\ \times \left[1-\tilde{\psi }(k)\left(1-\tilde{\alpha }\Theta (t)-\tilde{\beta }p(t)\right)\right]+o(p(t)). \end{array}$$ (B1)

Hence the Jacobian matrix of (2) at the DFE $\left(p^{*},q^{*}\right)$ is

$$J=\left[\begin{array}{cc} P & 0\\ R & Q \end{array}\right],$$ (B2)

where $P={\left(p_{kk\prime }\right)}_{n\times n}$ with

$$p_{kk\prime }=-\gamma \delta_{kk\prime }+\lambda \psi (k)(k-\alpha )(1-q_{k}^{*})\frac{k\prime P(k\prime )}{\left\langle k\right\rangle },$$ (B3)

$\delta_{kk\prime }=1$ if k=k′, otherwise $\delta_{kk\prime }=0$, and

$$Q=-[\delta +(1-\tilde{\psi }(k))]I,$$

and ***I***, **0**, are the identity and zero matrices of size *n* × *n*, respectively. Clearly, $\lambda_{\max}(J)=\max\{\lambda_{\max}(P),\;\lambda_{\max}(Q)\}$ where $\lambda_{\max}(\cdot )$ takes the maximum real parts of the eigenvalues of a matrix. Define $u:={\left(u_{1},u_{2},\ldots ,u_{n}\right)}^{T}$ and $v:={\left(v_{1},v_{2},\ldots ,v_{n}\right)}^{T}$ with $u_{k}:=\psi (k)(k-\alpha )(1-q_{k}^{*})$ and $v_{k}:=\frac{kP(k)}{\left\langle k\right\rangle }$. Then, by (B3), we have that $P=-\gamma I+\lambda uv^{T}$. And so, $\lambda_{\max}(P)=-\gamma +\lambda u^{T}v=-\gamma +\lambda \sum_{k}\psi (k)(k-\alpha )(1-q_{k}^{*})\frac{kP(k)}{\left\langle k\right\rangle }$. On the other hand, since $\delta ,\tilde{\psi }(k)\in [0,1]$, we have that $\lambda_{\max}(Q)=-\min_{k}[\delta +(1-\tilde{\psi }(k))]<0$. Hence the DFE $\left(p^{*},q^{*}\right)$ of model (2) is stable if and only if $\lambda_{\max}(P)<0$, or equivalently, $\frac{\lambda }{\gamma }<{\hat{\lambda }}_{c}$, where ${\hat{\lambda }}_{c}$ is defined as in (3). Moreover, since the epidemic disease breaks out when the stability of DFE of model (2) is lost, ${\hat{\lambda }}_{c}$ is indeed the threshold for the outbreak of epidemic disease.

To complete the proof, we note that, in the case of *δ* = 0, $\tilde{\alpha }=\tilde{\beta }=0$ and $\tilde{\psi }(k)\equiv 1$, we have that, by (2)

$$\begin{array}{c} {\dot{p}}_{k}(t)=-\gamma p_{k}(t)+[1-p_{k}(t)-q_{k}(0)]\\ \left\{\lambda \psi (k)\left(1-\beta p(t)\right)\Theta (t)\left[k(1-\alpha \Theta (t))-\alpha (1-\Theta (t))\right]\right\},\\ q_{k}(t)\equiv q_{k}(0). \end{array}$$

Hence, $q^{*}=q(0)$. The proof is complete. □

*Proof of Theorem 2*. In the case that $P(k)=k^{-r}/c,k=m,\ldots ,M$, where $c=\sum_{k=m}^{M}k^{-r}$, and functions *ψ* and $\tilde{\psi }$ are defined as in (6), we compute, via (5), that

$$\begin{array}{cc} {\hat{\lambda }}_{c} & =\frac{\left\langle k\right\rangle }{\sum_{k=m}^{M}\frac{\delta }{\delta +(1-k^{-\tilde{b}})}k^{-b}(k^{2}-\alpha k)(k^{-r}/c)}\\ & =\frac{\sum_{k=m}^{M}k^{1-r}}{\sum_{k=m}^{M}\frac{\delta (k^{2}-\alpha k)}{(\delta +1)k^{b+r}-k^{b+r-\tilde{b}}}}=:{\hat{\lambda }}_{c(SF)}. \end{array}$$ (B4)

Since the numerator of the above equation is finite for any r∈(2,3] and the denominator goes to ∞ as *M* tends to ∞ if and only if b+r≤3, the proof of theorem is complete. □

In Theorems 3–5, we aim to compare the size of ${\hat{\lambda }}_{c(SF)}$ and ${\hat{\lambda }}_{c(Homo)}$. To this end, we need to apply some well-known results, which are stated in the following two lemmas for ease of the references.

Lemma B.1 (Jesen's inequality). Let *P*(*k*) be a discrete probability distribution and *f* be a continuous function defined on [m,M]. If *f* is convex (respectively, concave) in [m,M], then $f\left(\sum_{k}x_{k}P(k)\right)\leq$ (respectively, ≥) $\sum_{k}f(x_{k})P(k)$. Moreover, both of the above equalities hold if and only if *x_k_* are all equal or *f* is linear.

Lemma B.2 (Theorem F in p. 19 of Ref. 46). Let *f* be a positive function defined in an interval *I*. Then *f* is said to be a log-convex function in *I* if ln(f) is convex in *I*. The class of log-convex functions in *I* is closed under addition, multiplication, and taking the limits, provided that the limits exist and are positive.

Proposition B.2. Define

$${\hat{\psi }}_{c}(x)=\frac{\delta }{\delta +(1-x^{-\tilde{b}})}x^{-b}(x^{2}-\alpha x).$$ (B5)

Then

(i) ${\hat{\lambda }}_{c(SF)}\leq {\hat{\lambda }}_{c(Homo)}$, whenever ${\hat{\psi }}_{c}(x)$ is convex in [m,M],
(ii) ${\hat{\lambda }}_{c(SF)}\geq {\hat{\lambda }}_{c(Homo)}$, whenever ${\hat{\psi }}_{c}(x)$ is concave in [m,M].

Moreover, both of the above equalities hold if and only if $\left(b,\tilde{b},\alpha \right)$ equals to $\left(1,0,a),\;(1,1,\frac{1}{\delta +1}\right)$, (2, 0, 0) or $\left(2,1,\frac{1}{\delta +1}\right)$ where a∈[0,1].

*Proof*. From (B4) and (7), it is clear that

$${\hat{\lambda }}_{c(SF)}=\frac{\left\langle k\right\rangle }{\sum_{k}{\hat{\psi }}_{c}(k)P(k)},$$

where $P(k)=k^{-r}/c$, and

$${\hat{\lambda }}_{c(Homo)}=\frac{\left\langle k\right\rangle }{{\hat{\psi }}_{c}(\langle k\rangle )},$$

respectively. Then by applying Lemma B.1 to function ${\hat{\psi }}_{c}$ and letting *x_k_* = *k*, the proof of the theorem is complete. □

Lemma B.3. Suppose $\tilde{b}=0$. Then the following assertions hold.

(i) If 0≤b<1, then ${\hat{\lambda }}_{c(SF)}<{\hat{\lambda }}_{c(Homo)}$;
(ii) If *b* = 1, then ${\hat{\lambda }}_{c(SF)}={\hat{\lambda }}_{c(Homo)}\left(=\frac{\left\langle k\right\rangle }{\langle k\rangle -\alpha }\right)$;
(iii) If 1<b<2, then ${\hat{\lambda }}_{c(SF)}>{\hat{\lambda }}_{c(Homo)}$;
(iv) If *b* = 2, then ${\hat{\lambda }}_{c(SF)}\geq {\hat{\lambda }}_{c(Homo)}$ and the equality holds only when *α* = 0.
(v) If *b* > 2 and $\alpha <\frac{b-2}{b}$, then ${\hat{\lambda }}_{c(SF)}<{\hat{\lambda }}_{c(Homo)}$.

*Proof*. In the case of $\tilde{b}=0$, we compute directly by (B5) that

$${\hat{\psi }}_{c}^{\prime \prime }(x)=(1-b)[(2-b)x+\alpha b]x^{-1-b}.$$

It follows that ${{\hat{\psi }}^{\prime \prime }}_{c}(x)>0$ (respectively, < 0) in [1,∞) if *b* < 1 (respectively, 1<b<2). Hence, statements (i) and (iii) in the lemma hold true by Proposition B.2.

For *b* > 2, since $\underset{x\to \infty }{\lim}{\hat{\psi }}_{c}^{\prime \prime }(x)=+\infty$ and $\text{sgn}({\hat{\psi }}_{c}^{\prime \prime }(x))=\text{sgn}((b-2)x-\alpha b)$ is increasing in *x*, ${\hat{\psi }}_{c}^{\prime \prime }(x)$ has the same sign in [1,∞) if and only if $\text{sgn}({\hat{\psi }}_{c}^{\prime \prime }(1))=\text{sgn}((b-2)-\alpha b)=1$, or equivalently, $\alpha <\frac{b-2}{b}$. Hence, statement (v) holds true. The remaining statements (ii), (iv), and (v) in the lemma can be shown similarly and thus are omitted. □

We are now in the position to prove Theorems 3–5.

*Proof of Theorem 3*. In the following, we only give the proof for the case that M=∞. When M<∞, the proof can be obtained similarly.

For any $\alpha \in [0,1],\;\tilde{b}\geq 0$ and r∈(2,3], define function *g*(*b*) in (3−r,∞) by

$$g(b):=\ln\left({\left[{\hat{\lambda }}_{c(SF)}(b,\alpha )\right]}^{-1}\right)-\ln\left({\left[{\hat{\lambda }}_{c(Homo)}(b,\alpha )\right]}^{-1}\right).$$ (B6)

Then by Theorem 2 and Eq. (7), *g* is well defined for all r∈(2,3]. Moreover, when b=3−r, since ${\hat{\lambda }}_{c(SF)}=0$ and ${\hat{\lambda }}_{c(Homo)}>0$, we have that $\underset{b\to {\left(3-r\right)}^{+}}{\lim}g(b)=+\infty$. We now show that *g* is convex. Since, by (B4)

$${\left[{\hat{\lambda }}_{c(SF)}(b,\alpha )\right]}^{-1}=\frac{1}{\left\langle k\right\rangle }\sum_{k}\frac{\delta (k^{2}-\alpha k)(k^{-r}/c)}{\delta +(1-k^{-\tilde{b}})}k^{-b},$$

we compute hat $\frac{\partial^{2}}{\partial b^{2}}\ln\left[\frac{\delta (k^{2}-\alpha k)ck^{-r}}{\delta +(1-k^{-\tilde{b}})}k^{-b}\right]\equiv 0$. It implies that $\left[\frac{\delta (k^{2}-\alpha k)ck^{-r}}{\delta +(1-k^{-\tilde{b}})}k^{-b}\right]$ is a log-convex function. Consequently, ${\left[{\hat{\lambda }}_{c(SF)}(b,\alpha )\right]}^{-1}$ is also a log-convex function of *b* by Lemma B.2. On the other hand, since, by (7)

$${\left[{\hat{\lambda }}_{c(Homo)}(b,\alpha )\right]}^{-1}=\frac{1}{\left\langle k\right\rangle }\frac{\delta \left[{\left\langle k\right\rangle }^{2}-\alpha \langle k\rangle \right]}{\delta +[1-{\left\langle k\right\rangle }^{-\tilde{b}}]}{\left\langle k\right\rangle }^{-b},$$

we compute that $\frac{\partial^{2}}{\partial b^{2}}\ln\left[{\left[{\hat{\lambda }}_{c(Homo)}(b,\alpha )\right]}^{-1}\right]\equiv 0$. From the above, we conclude that $g^{\prime \prime }(b)\geq 0$ on (3−r,∞). That is, *g* is convex on (3−r,∞).

We next show the existence of $b_{2}(\alpha ,m,M)$ for α∈[0,1) and m∈ℕ in the case that $\tilde{b}=0$. From Lemma B.3, it is easy to see that, for any α∈[0,1), *g*(*b*) > 0 when $b\in [3-r,1)\cup (\frac{2}{1-\alpha },\infty ),\;g(1)=0$, while *g*(*b*) < 0 when b∈(1,2). Then the convex property of *g* implies that it has exactly two zeros at *b* = 1 and some $b_{2}\;(=b_{2}(\alpha ,m,M))\in [2,\frac{2}{1-\alpha })$. In fact, *g*(*b*) > 0 when $b\in [0,1)\cup (b_{2},\infty )$ and *g*(*b*) < 0 when $b\in (1,b_{2})$. Then by the definition of *g*, the inequalities stated in the theorem hold true.

Now, we show that $b_{2}(\alpha ,m,M)$ is strictly increasing in α∈[0,1). Suppose not, then there exist $\alpha_{1}\neq \alpha_{2}\in [0,1)$ such that $b_{2}(\alpha_{1},m,M)=b_{2}(\alpha_{2},m,M)\;(=:b^{*})$. By the definition of function $b_{2}(\alpha ,m,M)$, ${\hat{\lambda }}_{c(SF)}(b^{*},\alpha_{1})={\hat{\lambda }}_{c(Homo)}(b^{*},\alpha_{2})$ and $b^{*}\geq 2$ as claimed above. For each fixed b>3−r, define $h_{b}(\alpha )$ in ${\mathbb{R}}^{+}$ by

$$\begin{array}{c} h_{b}(\alpha ):={\left[{\hat{\lambda }}_{c(SF)}(b,\alpha )\right]}^{-1}-{\left[{\hat{\lambda }}_{c(Homo)}(b,\alpha )\right]}^{-1}\\ =\left[\frac{\left\langle \frac{\delta k^{2-b}}{\delta +1-k^{-\tilde{b}}}\right\rangle -\frac{\delta }{\delta +1-{\left\langle k\right\rangle }^{-\tilde{b}}}{\left\langle k\right\rangle }^{2-b}}{\left\langle k\right\rangle }\right]\\ \;-\left[\frac{\left\langle \frac{\delta k^{1-b}}{\delta +1-k^{-\tilde{b}}}\right\rangle -\frac{\delta }{\delta +1-{\left\langle k\right\rangle }^{-\tilde{b}}}{\left\langle k\right\rangle }^{1-b}}{\left\langle k\right\rangle }\right]\alpha . \end{array}$$ (B7)

Then clearly, ${\hat{\lambda }}_{c(SF)}(b,\alpha )={\hat{\lambda }}_{c(Homo)}(b,\alpha )$ if and only if $h_{b}(\alpha )=0$. Hence, $h_{b^{*}}(\alpha_{1})=h_{b^{*}}(\alpha_{2})=0$. Note that in the case that $\tilde{b}=0$, function *h_b_* becomes

$$h_{b,\tilde{b}=0}(\alpha )=\left[\frac{\langle k^{2-b}\rangle -{\left\langle k\right\rangle }^{2-b}}{\left\langle k\right\rangle }\right]-\left[\frac{\langle k^{1-b}\rangle -{\left\langle k\right\rangle }^{1-b}}{\left\langle k\right\rangle }\right]\alpha .$$

Since, by Lemma B.1, $\langle k^{2-b}\rangle \geq {\left\langle k\right\rangle }^{2-b}$ for b≥2 and the equality holds if and only if *b* = 2, we have that $\langle k^{1-b^{*}}\rangle -{\left\langle k\right\rangle }^{1-b^{*}}>0$. It follows that $h_{b^{*}}$ has exactly one zero, which is a contradiction to the fact that $h_{b^{*}}(\alpha_{1})=h_{b^{*}}(\alpha_{2})=0$. Moreover, it is clear to see that, since $h_{b^{*}}(\alpha )=0$, we have that:

$$\frac{\langle k^{2-b_{2}^{*}}\rangle -{\left\langle k\right\rangle }^{2-b_{2}^{*}}}{\langle k^{1-b_{2}^{*}}\rangle -{\left\langle k\right\rangle }^{1-b_{2}^{*}}}=\alpha .$$

Hence, the proof is complete. □

*Proof of Theorem 4*. Note that the facts that the function *g*(*b*), defined in (B6), is convex on (3−r,∞) and $\lim_{b\to {\left(3-r\right)}^{+}}g(b)=+\infty$ are proved for all $\tilde{b}\geq 0$ and α∈[0,1]. Moreover, by Theorem 2 and Eq. (7), *g*(*b*) > 0 for *b* on (3−r,∞) or there exists some nonempty open interval *J* such that *g*(*b*) < 0 if and only if b∈J. These two scenarios correspond to ${\hat{\lambda }}_{c(SF)}<{\hat{\lambda }}_{c(Homo)}$ and ${\hat{\lambda }}_{c(SF)}>{\hat{\lambda }}_{c(Homo)}$, respectively.

On the other hand, consider the function $h_{b}(\alpha )$ defined in (B7). Then ${\hat{\lambda }}_{c(SF)}(b,\alpha )={\hat{\lambda }}_{c(Homo)}(b,\alpha )$ if and only if $h_{b}(\alpha )=0$ or equivalently, *b* satisfies

$$\frac{\left\langle \frac{\delta k^{2-b}}{\delta +1-k^{-\tilde{b}}}\right\rangle -\frac{\delta }{\delta +1-{\left\langle k\right\rangle }^{-\tilde{b}}}{\left\langle k\right\rangle }^{2-b}}{\left\langle \frac{\delta k^{1-b}}{\delta +1-k^{-\tilde{b}}}\right\rangle -\frac{\delta }{\delta +1-{\left\langle k\right\rangle }^{-\tilde{b}}}{\left\langle k\right\rangle }^{1-b}}=\alpha .$$

Hence, the proof is complete. □

*Proof of Theorem 5*. For $\tilde{b}>0$, we compute directly that

$${\hat{\psi }}_{c}^{\prime \prime }(x)=\frac{\delta x^{-2\tilde{b}-b-1}}{{\left[\delta +(1-x^{-\tilde{b}})\right]}^{3}}\;\overset{ˇ}{\psi }(x),$$

where

$$\begin{array}{cc} \overset{ˇ}{\psi }(x) & =(b-1)(b-2){\left(\delta +1\right)}^{2}x^{2\tilde{b}+1}-\alpha b(b-1){\left(\delta +1\right)}^{2}x^{2\tilde{b}}\\ & \;-(2b^{2}-2b\tilde{b}-{\tilde{b}}^{2}-6b+3\tilde{b}+4)(\delta +1)x^{\tilde{b}+1}\\ & \;+\alpha (2b^{2}-2b\tilde{b}-{\tilde{b}}^{2}-2b+\tilde{b})(\delta +1)x^{\tilde{b}}\\ & \;+(b-\tilde{b}-1)(b-\tilde{b}-2)x-\alpha (b-\tilde{b})(b-\tilde{b}-1). \end{array}$$

Then $\text{sgn}({\hat{\psi }}_{c}^{\prime \prime }(x))=\text{sgn}(\overset{ˇ}{\psi }(x))$. Clearly, when b∈[0,1)∪(2,∞), we have that $\lim_{x\to \infty }\overset{ˇ}{\psi }(x)=+\infty$, which implies that there exists some positive integer *m*_0_ such that $\overset{ˇ}{\psi }(x)>0$ for $x\geq m_{0}$. Hence, by Proposition B.2, ${\hat{\lambda }}_{c(SF)}<{\hat{\lambda }}_{c(Homo)}$ whenever the minimum degree in the SF network is greater than *m*_0_. On the other hand, when b∈(1,2), since $\lim_{x\to \infty }\overset{ˇ}{\psi }(x)=-\infty$, ${\hat{\lambda }}_{c(SF)}>{\hat{\lambda }}_{c(Homo)}$ whenever the minimum degree in the SF network is greater than some positive integer *m*_0_. The remaining cases stated in the Theorem can be proved similarly. □
